# Holographic Ultrasound Modulates Neural Activity in a 1-Methyl-4-Phenyl-1,2,3,6-Tetrahydropyridine-Induced Mouse Model of Parkinson’s Disease

**DOI:** 10.34133/research.0516

**Published:** 2024-11-06

**Authors:** Hui Zhou, Fei Li, Zhengrong Lin, Long Meng, Dan Chen, Qingping Zhang, Lili Niu

**Affiliations:** ^1^ Paul C. Lauterbur Research Center for Biomedical Imaging, Institute of Biomedical and Health Engineering, Shenzhen Institutes of Advanced Technology, Chinese Academy of Sciences, Shenzhen, China.; ^2^ Tech X Academy, Shenzhen Polytechnic University, Shenzhen, China.; ^3^ Institute of Ultrasonic Technology, Institute of Intelligent Manufacturing Technology, Shenzhen Polytechnic University, Shenzhen, China.; ^4^ School of Electronic and Communication Engineering, Shenzhen Polytechnic University, Shenzhen, China.

## Abstract

Ultrasound (US) has emerged as a noninvasive neurostimulation method for motor control in Parkinson’s disease (PD). Previous in vivo US neuromodulation studies for PD were single-target stimulation. However, the motor symptoms of PD are linked with neural circuit dysfunction, and multi-target stimulation is conducted in clinical treatment for PD. Thus, in the present study, we achieved multi-target US stimulation using holographic lens transducer based on the Rayleigh–Sommerfeld diffraction integral and time-reversal methods. We demonstrated that holographic US stimulation of the bilateral dorsal striatum (DS) could improve the motor function in 1-methyl-4-phenyl-1,2,3,6-tetrahydropyridine (MPTP)-induced mouse model of PD. The holographic US wave (fundamental frequency: 3 MHz, pulse repetition frequency: 500 Hz, duty cycle: 20%, tone-burst duration: 0.4 ms, sonication duration: 1 s, interstimulus interval: 4 s, spatial-peak temporal-average intensity: 180 mw/cm^2^) was delivered to the bilateral DS 20 min per day for consecutive 10 d after the last injection of MPTP. Immunohistochemical c-Fos staining demonstrated that holographic US significantly increased the c-Fos-positive neurons in the bilateral DS compared with the sham group (*P* = 0.003). Moreover, our results suggested that holographic US stimulation of the bilateral DS ameliorated motor dysfunction (*P* < 0.05) and protected the dopaminergic (DA) neurons (*P* < 0.001). The neuroprotective effect of holographic US was associated with the prevention of axon degeneration and the reinforcement of postsynaptic densities [growth associated protein-43 (*P* < 0.001), phosphorylated Akt (*P* = 0.001), β3-tubulin (*P* < 0.001), phosphorylated CRMP2 (*P* = 0.037), postsynaptic density (*P* = 0.023)]. These data suggested that holographic US-induced acoustic radiation force has the potential to achieve multi-target neuromodulation and could serve as a reliable tool for the treatment of PD.

## Introduction

Parkinson’s disease (PD) is the second most common neurodegenerative disorder following Alzheimer’s disease around the world [1]. The most important pathological feature of PD is the loss of dopaminergic (DA) neurons in the substantia nigra (SN) [2,3]. The clinical symptoms of PD include resting tremor, bradykinesia, muscular rigidity, and slow of voluntary movements [4]. These motor symptoms occur when the loss of DA neurons in the SN reaches about 50% to 70% [5]. In addition, as the disease advances, these motor-related symptoms become increasingly pronounced [6]. PD severely affects the quality life of patients and brings huge economic challenges to patients, their families, and the society [7].

Physical tools, such as transcranial magnetic stimulation (TMS) [8], deep brain stimulation (DBS) [9], transcranial direct current stimulation (tDCS) [10], and low-intensity ultrasound (US) [11], have been used to modulate the neural activities. Furthermore, bilateral stimulation of the brain nuclei has been widely used for the treatment of neural circuit dysfunction and neurological diseases in clinical trials [12–14]. Bilateral DBS of the subthalamic nucleus (2 targets) achieved better amelioration in motor performance, compared with unilateral target stimulation in patients with severe PD [15,16]. The clinical US treatment for PD includes blood–brain barrier (BBB) opening with focused US for drug delivery [17–19], high-intensity focused ultrasound (HIFU) for ablation [20–22], and low-intensity US for neuromodulation [23–25]. Focused US combined with microbubbles could enhance the permeability of the BBB and facilitate drug delivery locally to the putamen for PD patients [26]. Bilateral HIFU ablation of the pallidothalamic tract improved parkinsonian-related dyskinesia [27,28]. Compared with BBB opening and HIFU ablation, low-intensity US neuromodulation gains increasing attention due to its drug-free and reversible effect.

Low-intensity US treatment for PD has made some progresses in animal experiments and clinical trials. Wang et al. [29] found that transcranial US stimulation of the motor cortex inhibited parkinsonian-related neural activity in mouse model of PD. We also demonstrated that US deep brain stimulation effectively improved the motor symptoms in PD mice [30,31]. Samuel et al. [25] found that 2 array transducers stimulation of the bilateral motor cortex enhanced the motor cortex excitability in PD patients. However, the bilateral motor cortex stimulation effect on the motor performance in their study needs further validation. Several issues exist in current bilateral US neuromodulation for PD, including the lack of portable tools for bilateral stimulation, refined treatment targets, and the underlying mechanisms. Preclinical animal experiments would help to elucidate these issues.

US neurostimulation experiments in animal models have been limited to single-spot sonication because of the size and weight of US transducer. Jin et al. [32] fabricated a needle transducer with 1.6 mm diameter, which could conduct multi-target stimulation in mice. However, the depth of the transducer was approximately 1 mm and could not be used for deep brain US stimulation. Zhang et al. [33] performed dual-target US neuromodulation in mice using a 2-dimensional (2D) array transducer with 256 elements. Li et al. [34] have indicated that array system could support multiple-target stimulation by allocating multiple focal points in the brain. However, this required complicated electronic control system and incurred considerable economical costs [35]. He et al. [36] fabricated a bifocal holographic lens for bilateral US stimulation. However, the treatment effects of holographic US in PD mice and the underlying mechanisms have not been elucidated.

In this study, we designed a bifocal holographic lens based on the Rayleigh–Sommerfeld diffraction integral and time-reversal methods. We combined the holographic lens with a wearable transducer for free moving mouse. The holographic transducer worked at 3 MHz, with 10 mm × 10 mm length and 2 mm height. The bifocal beams generated by the holographic lens match with the location of the bilateral dorsal striatum (DS) of mouse. We further explored the feasibility of using the holographic transducer for US neuromodulation in PD mice. The c-Fos staining confirmed that holographic stimulation evoked neural activation in the DS. A subacute 1-methyl-4-phenyl-1,2,3,6-tetrahydropyridine (MPTP) mouse model of PD was built to verify the treatment effect of holographic US stimulation. The behavioral tests indicated that holographic US improved motor function in the pole and rotarod tests. Correspondingly, holographic US significantly increased the number of the DA neurons in the SN by alleviating axon degeneration and promoting postsynaptic densities. These studies demonstrated that the DS may serve as a novel target for ultrasonic treatment of PD. Moreover, we introduced a holographic transducer as an alternative way to reconstruct acoustic field for US neuromodulation.

## Results

### Lens design and transducer fabrication

**Fig. 1.**
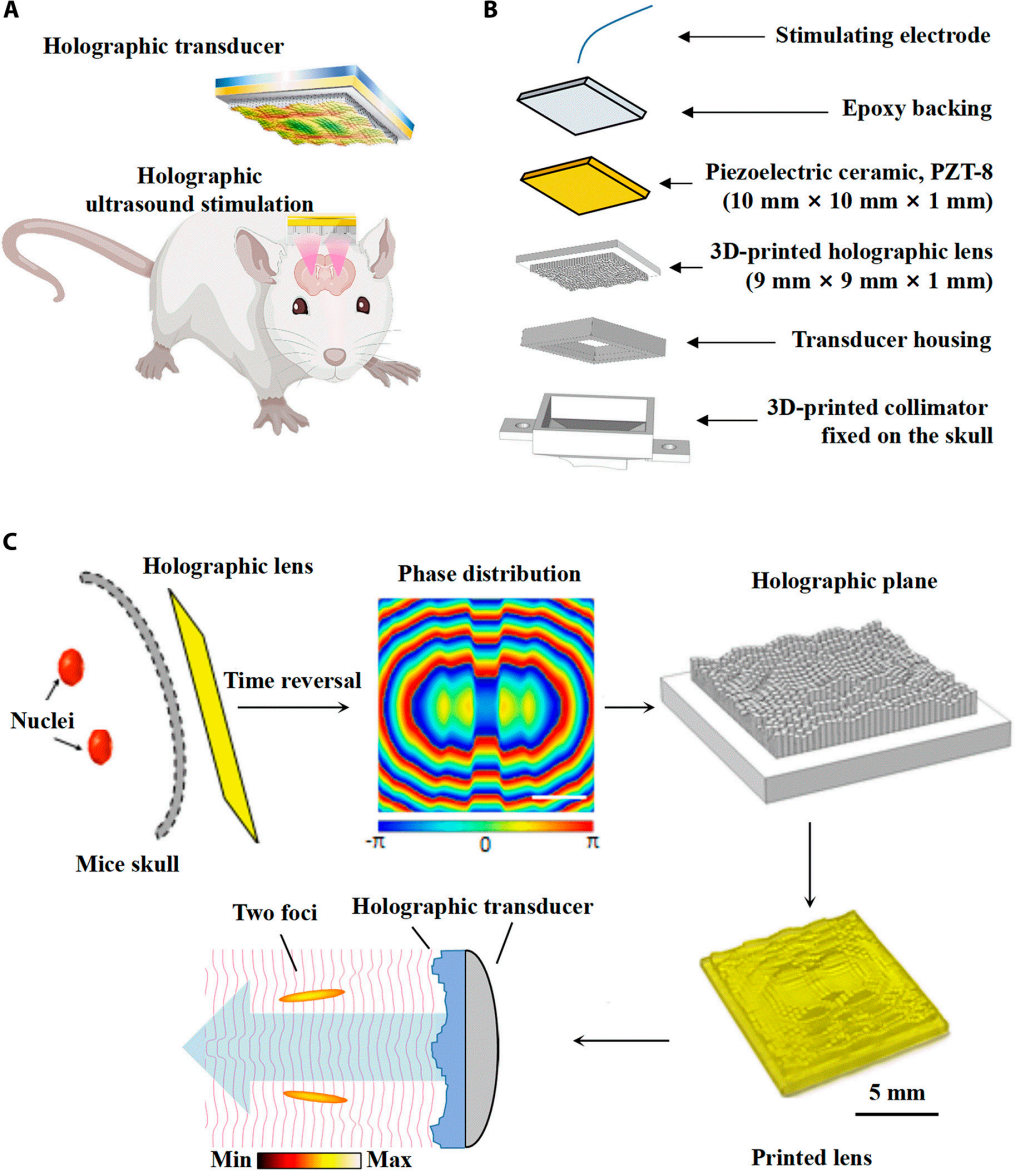
Schematic diagram and design of the holographic lens and holographic transducer. (A) Schematic diagram of the holographic US for bilateral stimulation. (B) Assembly of holographic transducer for freely moving mice. (C) Lens design process: the bilateral striatum of the mouse was selected as 2 virtual acoustic sources (red area), mouse skull (gray area), and holographic recording surface (yellow area). The holographic lens converted the planar wavefront from a single-element transducer into a bifocal distribution.

The proposed multi-target holographic lens possessed a remarkable property that enabled acoustic phase compensation caused by the mouse skull. In this study, the time-reversal method was used to calculate the holographic beam bending following the target path, and the phase and pressure intensity distributions in the holographic plane. The bilateral DS of mouse was selected as 2 virtual acoustic sources (Fig. 1C, red area). The acoustic pressure field *p*(**d**, *ω*) at point **d** in the holographic plane of the holographic beam, generated by a moving surface S of arbitrary shape at coordinates **d**_**0**_, could be calculated using the Rayleigh–Sommerfeld diffraction integral [37], as follows:

$$p\left(d,\omega \right)=\frac{i\omega \rho_{0}}{2\pi }\int_{S}\frac{u_{0}\left(d_{0}\right)\text{exp}\left(-ik_{0}\left|d-d_{0}\right|\right)}{\left|d-d_{0}\right|}dS$$ (1)

where *u_0_*(**d**_0_) denotes the particle vibration velocity of the moving surface S, and *ω* = 2*πf*, *f* denotes the frequency of the US wave, and *k*_0_ = *ω*/*c*_0_, *c*_0_, and *ρ*_0_ denoted the wave number, sound speed, and density of water, respectively. The phase field *ϕ*(**d**, *ω*) in the holographic plane at point **d** was given by:

$$\phi \left(d,\omega \right)=\text{arg}\left(p\left(d,\omega \right)\right)=\text{arctan}\frac{\text{Im}\left(p\left(d,\omega \right)\right)}{\text{Re}\left(p\left(d,\omega \right)\right)}$$ (2)

where Im(*p*(**d**, *ω*)) and Re(*p*(**d**, *ω*)) denote the imaginary and real part of the pressure *p*(**d**, *ω*), respectively.

To design the holographic lens with bifocus, 2 set 100 virtual sources were located at the position of the DS, each source compensated by a phase factor of exp(*ik_z_z*) accounting for the direction of arrival of the wavefront. The recorded field of phase was captured at a given surface, i.e., at a holographic plane, outside the mouse skull, as shown in Fig. 1C (yellow area).

The recorded phase distribution at the working frequency was used to design the holographic lens. The lens surface was divided in square pixels of different height, *h*(*x*, *y*), and uniform width, Δ*w* = 0.25 mm. For each square pixel, the pressure field at the holographic lens surface located at **r**_**0**_(*x*, *y*, *l*) is given by the complex transmission coefficient [37]:

$$T\left(r_{0}\right)=\frac{2Ze^{-ik_{0}\left[l-h\left(r_{0}\right)\right]}}{2Z\text{cos}\left[k_{L}h\left(r_{0}\right)\right]+\;i\left(Z^{2}+1\right)\text{sin}\left[k_{L}h\left(r_{0}\right)\right]}$$ (3)

where *Z* is the normalized impedance given by *Z* = *Z_L_*/*Z*_0_; *Z*_0_ = *ρ*_0_*c*_0_ is the impedance of water; and *Z_L_* = *ρ_L_c_L_*, *k_L_* = *ω*/*c_L_*, *ρ_L_*, and *c_L_* are the impedance, wave number, density, and sound speed of the lens material. *l* is the distance from the bottom of the lens (*z* = 0) to the holographic lens surface. The phase field at the holographic lens surface is given by:

$$\varphi \left(r_{0}\right)=\text{arg}\left(T\left(r_{0}\right)\right)=\text{arctan}\frac{\text{Im}\left(T\left(r_{0}\right)\right)}{\text{Re}\left(T\left(r_{0}\right)\right)}$$ (4)

where Im(*T*(**r**_0_)) and Re(*T*(**r**_0_)) denote the imaginary and real part of the pressure *T*(**r**_**0**_), respectively. Combined with the acoustic phase fields calculated by the above equations, the height *h*(*x*, *y*) of the pixels of the holographic lens was obtained. Then, the holographic lens was numerically designed and afterward manufactured using a 3D-printing stereolithographic technique (Formlabs, USA) with a resolution of 100 μm in both lateral and axial directions and using a photosensitive resin (Standard Clear, Formlabs, USA).

In this study, the finite element method (FEM) was used to simulate the acoustic pressure fields. The acoustic properties of the water, holographic lens, and skull were given in Table [38]. The thickness of the skull was 250 μm in numerical simulation. All materials were assumed to be ideal, homogeneous, and linearly elastic without attenuation. A plane wave of unit amplitude was impinged on the bottom of the holographic lens and converted into a bifocal distribution.

**Table.** Acoustic properties of the water, lens, and skull

	Density (kg/m^3^)	Longitudinal velocity (m/s)	Transversal velocity (m/s)
Water	1,000	1,500	Null
Lens	1,180	2,700	1,160
Skull	1,912	2,300	1,626

**Fig. 2.**
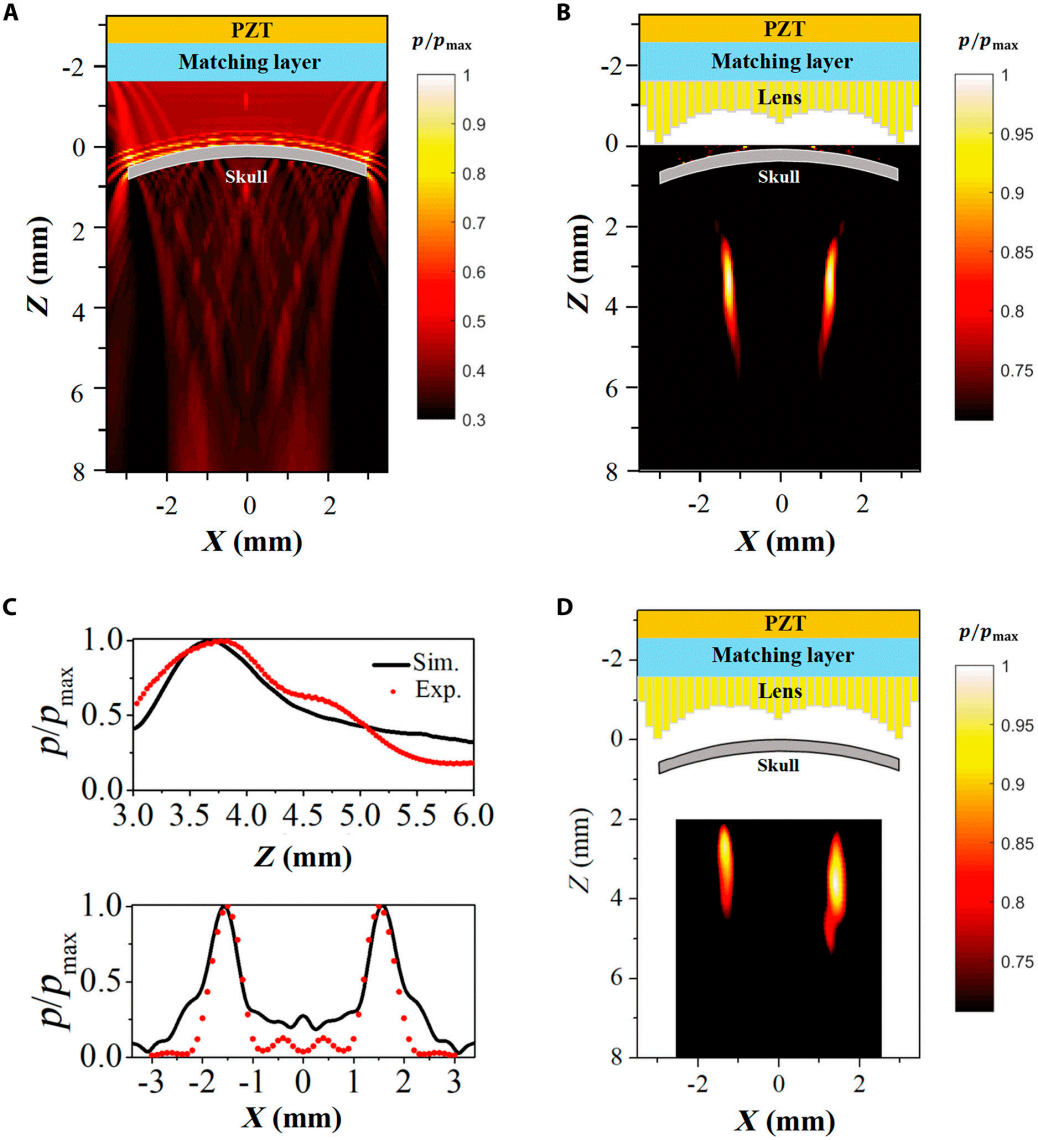
An acoustic field distribution modulated by the holographic lens. Numerically simulated pressure fields after passing the mouse skull without (A) and with holographic lens (B). (C) Simulated and measured acoustic pressure distribution along the line *x* = 1.5 mm, *y* = 0, *z* = [2.5 mm, 5.5 mm] (top) and the line *x* = [−2.5 mm, 2.5 mm], *y* = 0, *z* = 3.5 mm (bottom), respectively. (D) Experimentally measured pressure field after passing the mouse skull with holographic lens.

The holographic transducer for free moving mouse was shown in Fig. 1A and B. The assembly of the holographic transducer (Fig. 1B) comprised a stimulating electrode, epoxy backing, piezoelectric ceramic, and 3D-printed holographic lens transducer housing. The bifocal region of the transducer, after passing the mice skull, was matched with the bilateral striatum of the mice (Fig. 2D and Fig. S1E).

### Acoustic pressure distribution

The numerically calculated and experimentally measured pressure fields produced by the planar US transducer without a lens in the *x*-*z* plane were shown in Fig. 2A. The holographic lens was placed in front of the planar US transducer, and a 2-focus field was formed at *z* = 3.5 mm, as shown in Fig. 2B. The experimental results were consistent with the simulation results, as shown in Fig. 2D. We then quantitatively compared the pressure distribution between the simulated (displayed by black solid lines) and the measured results (displayed by red dots) along the line *x* = 1.5 mm, *y* = 0, *z* = [2.5 mm, 5.5 mm] and the line *x* = [−2.5 mm, 2.5 mm], *y* = 0, *z* = 3.5 mm, respectively, as shown in Fig. 2C. The measured data were in good agreement with the simulated results. The asymmetry of the left and right experimental focal spots was caused by an uneven skull, as shown in Fig. 2D, demonstrating that the transcranial sound field designed using the time-reversal calculation method was little affected by the skull [37]. The numerical simulated pressure field of the holographic transducer with and without skull was shown in Fig. S1B and C. Wei et al. [39] have indicated that the murine skull changes with age, ranging from 100 to 300 μm. To investigate how the thickness of mice skull affects the acoustic field, we conducted simulations for focused acoustic beams through skulls with thicknesses ranging from 100 to 300 μm in increments of 100 μm, as shown in Fig. S2. According to the simulation results, the thickness of skull had little influence on the pressure field after modulation by the holographic lens.

### Holographic US stimulation of the bilateral striatum enhanced the c-Fos expression in the DS

**Fig. 3.**
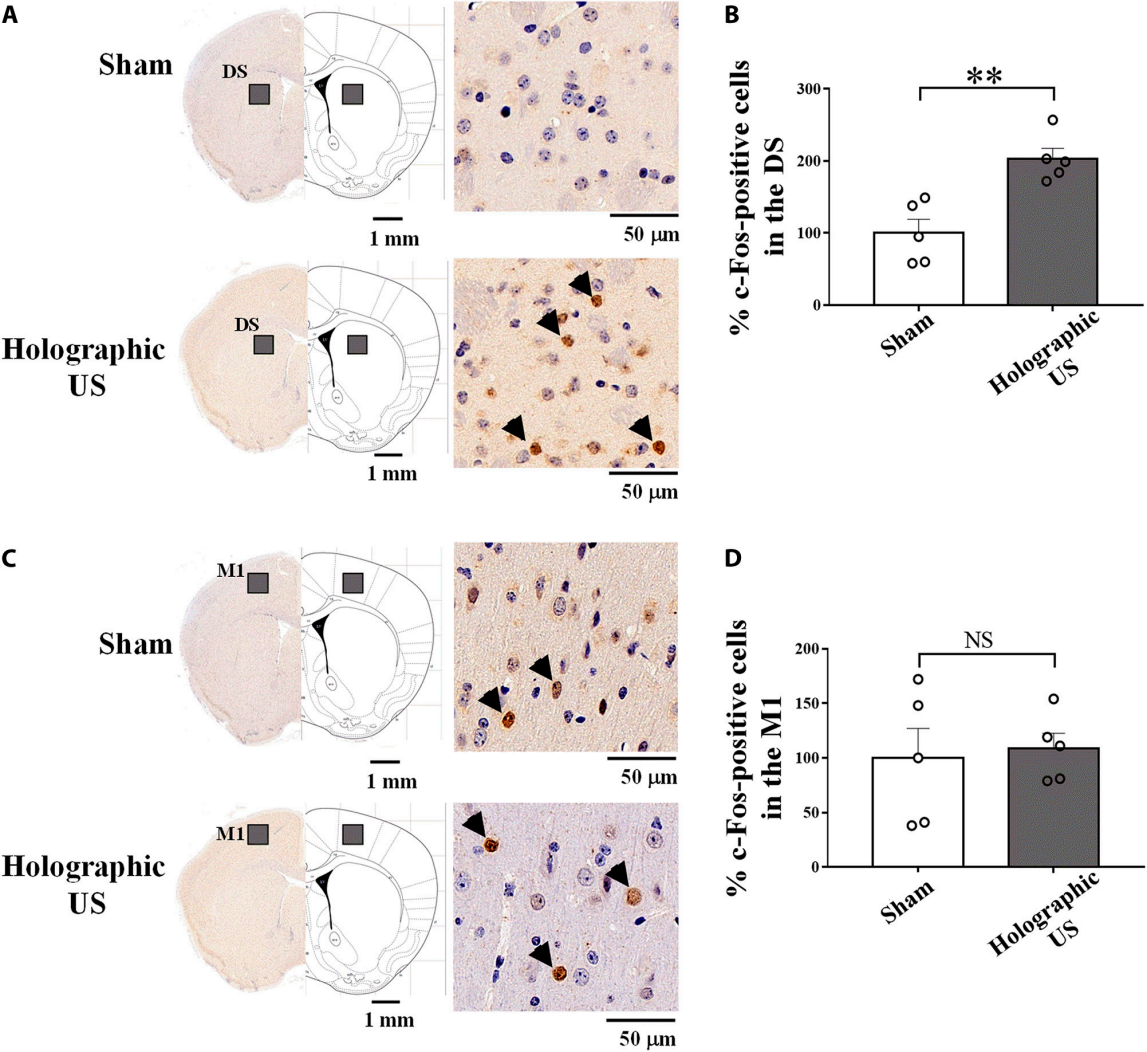
Increase in the neuronal activities in the DS, but not in M1 induced by holographic US. Representative images of c-Fos (marked by blown arrows) staining in the DS (A) and M1 (C). Quantification of c-Fos-positive cells in the DS (B) and M1 (D) after holographic US stimulation, compared with the sham group. The holographic US increased c-Fos expression in the DS, but not in M1 (independent-sample *t* test, mean ± SEM, *n* = 5 per group).

We examined whether the holographic US could activate neural activities in vivo. Healthy mice were randomly allocated into 2 groups: the sham group (*n* = 5) and the holographic US group (*n* = 5). Mice in the holographic US group received bilateral US stimulation of the DS for 30 min. The holographic US wave (Fig. S3) was follows: fundamental frequency (FF): 3 MHz, pulse repetition frequency (PRF): 500 Hz, duty cycle (DC): 20%, tone-burst duration (TBD): 0.4 ms, sonication duration (SD): 1 s, interstimulus interval (ISI): 4 s, spatial-peak temporal-average intensity: 180 mw/cm^2^. After 90 min for full expression, c-Fos staining was performed to monitor the number of c-Fos-positive cells in the DS and primary motor cortex (M1), respectively. Holographic US stimulation resulted in a specific increase in the number of c-Fos-positive cells in the DS (sham: 100 ± 18%, holographic US: 203 ± 15%, *P* = 0.003; Fig. 3A). These results demonstrated that holographic US facilitated neural activity in the DS. Primary motor cortex (M1) is also involved in basal ganglia circuit. Studies have already shown that US stimulation of M1 led to improvements in motor function of PD mice [29,40]. Thus, we assessed the neural activity in M1 to evaluate whether holographic US induced any neural activity in M1. As illustrated in Fig. 3C, there was no significant difference observed in M1 after bilateral US stimulation (sham: 100 ± 27%, holographic US: 109 ± 14% per mm^2^, *P* = 0.782). Consequently, we excluded the potential M1 effect on the motor performance in mice with PD.

### Holographic US stimulation of the bilateral striatum improved the motor performance of PD mice

**Fig. 4.**
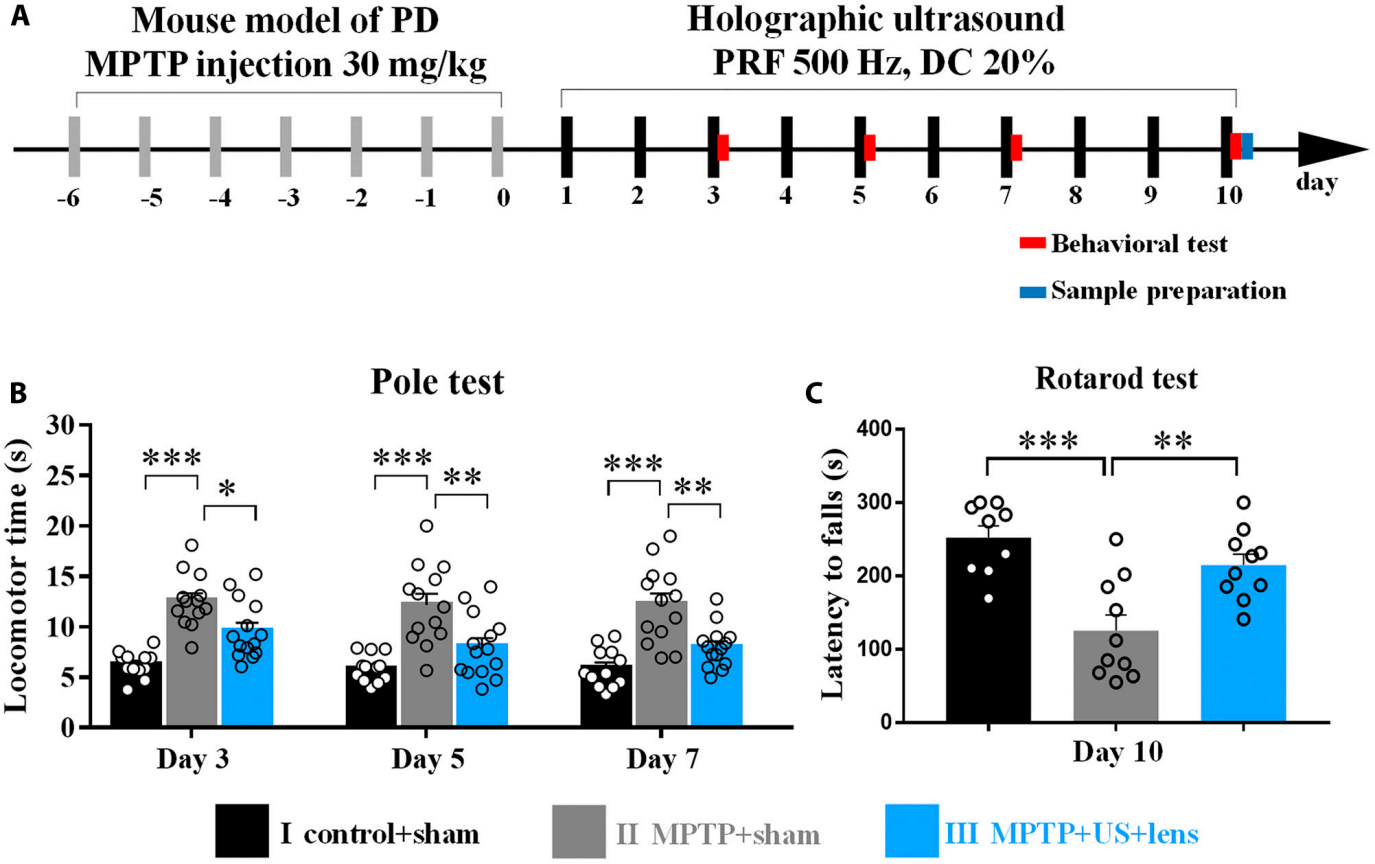
Holographic US stimulation improved the motor function of PD mice. (A) Illustration of behavior test protocol. After 7-d injection of MPTP, mice received holographic US stimulation for 10 d. During the US stimulation, the behavioral tests were performed on days 3, 5, 7, and 10. (B) Holographic US stimulation recovered the locomotor time in the pole test. The locomotor time was decreased in group III after holographic US treatment. (C) Holographic US stimulation recovered the motor behavior in the rotarod test. The latency time was increased after holographic US stimulation in PD mice. *P < 0.05, **P < 0.01, ***P < 0.001, mean ± SEM; one-way ANOVA with Bonferroni’s post hoc, *n* = 12 for group I, *n* = 13 for group II, *n* = 14 for group III in the pole test; one-way ANOVA with Tukey’s post hoc, *n* = 9 for group I, *n* = 10 for group II, *n* = 10 for group III in the rotarod test.

We then assessed the motor performance of PD mice using the pole and rotarod tests. Mice were divided into 3 groups: (I) control + sham, (II) MPTP + sham, and (III) MPTP + US + lens (holographic US stimulation). During the preparation stage (day −6 to day 0), mice received MPTP injection (30 mg/kg, Sigma-Aldrich) for 7 consecutive days to induce parkinsonism symptoms, and group I mice received an equivalent volume of saline. During the treatment stage (day 1 to day 10), mice received holographic US stimulation (3 MHz FF, 500 Hz PRF, 20% DC, 0.4 ms TBD, 1 s SD, 4 s ISI) for 10 d. Holographic US was delivered to the bilateral DS (Fig. S1E) daily for 10 d. The pole and rotarod tests were adopted to assess the behavioral performance of mice. As shown in Fig. 4B, the locomotor time was significantly increased in group II, while the group I mice maintained normal motor functions on day 3 (group I: 6.29 ± 0.37 s, group II: 12.59 ± 0.73 s, *P* < 0.001), day 5 (group I: 5.91 ± 0.39 s, group II: 12.17 ± 1.09 s, *P* < 0.001), and day 7 (group I: 5.92 ± 0.55 s, group II: 12.24 ± 1.07 s, *P* < 0.001). After holographic US stimulation, the locomotor time in group III was decreased as compared with that in group II on day 3 (group III: 9.64 ± 0.77 s, *P* = 0.021), day 5 (group III: 8.05 ± 0.83 s, *P* = 0.008), and day 7 (group III: 7.99 ± 0.56 s, *P* = 0.001). The latency time in the rotarod test was measured to evaluate the degree of motor dysfunction. As shown in Fig. 4C, the latency to falls was decreased in group II as compared with that in group I on day 10 (group I: 252.06 ± 16.19 s, group II: 125.45 ± 21.51 s, *P* < 0.001). After holographic US stimulation, the latency to falls was increased in group III as compared with group II on day 10 (group III: 214.95 ± 15.02 s, *P* = 0.004). These behavioral results indicated that holographic US stimulation of the bilateral striatum improved the motor performance of PD mice.

### Holographic US stimulation of the bilateral striatum protected the DA nigrostriatal pathway from MPTP toxicity

**Fig. 5.**
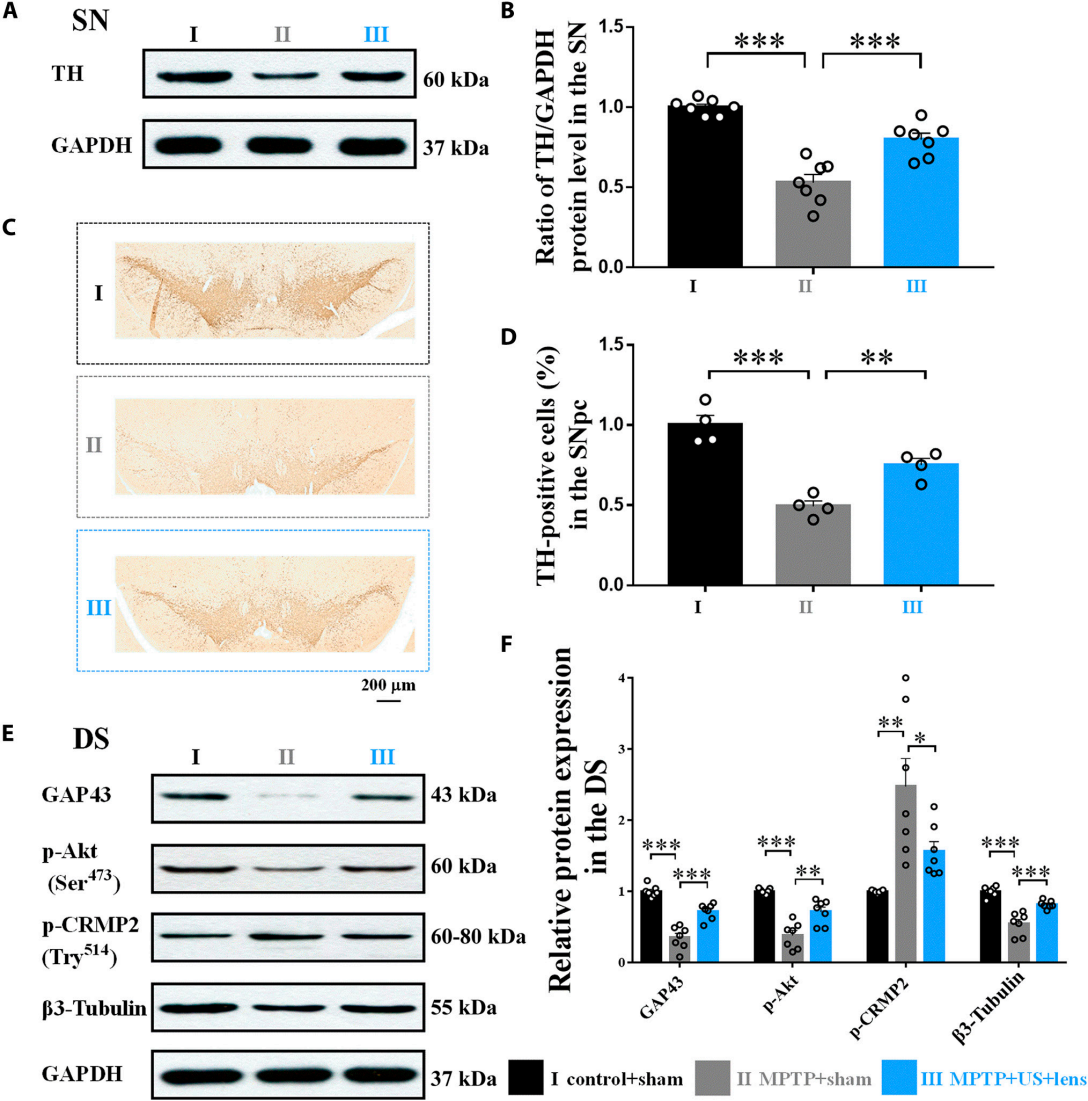
Holographic US provided neuroprotective effect for the axon degeneration. Holographic US increased the TH protein level (A and B) and DA neurons in the SN (C and D), as compared with the MPTP + sham group. Analysis of GAP-43, p-Akt, β3-tubulin, and p-CRMP2 expression in the DS (E). GAP-43, p-Akt, and β3-tubulin were increased and β3-tubulin was decreased in the DS after holographic US stimulation (F). One-way ANOVA with Tukey’s post hoc: *P < 0.05, **P < 0.01, ***P < 0.001; mean ± SEM, *n* = 7 for Western blot, *n* = 4 per group for TH immunohistochemistry.

The behavior performance of PD is closely linked with the number of DA neurons in the SN [41]. After observing the behavioral improvements induced by holographic US stimulation of the bilateral striatum, we explored whether holographic US could provide neuroprotective effect for DA neurons. After holographic US stimulation, all mice were sacrificed for immunohistochemistry and Western blotting (WB) analysis. The number of TH-positive cells was decreased in group II (group I: 1.00 ± 0.06, group II, 0.49 ± 0.03, *P* < 0.001; Fig. 5C and D), indicating the severe pathology of PD induced by MPTP injection. The mice in group III showed enhanced number of TH-positive cells in the SN compared with that in group II (group III: 0.75 ± 0.04, *P* = 0.009; Fig. 5C and D). The elevated TH protein level in the SN after holographic US stimulation of the bilateral striatum was further validated by WB analysis (group I: 1.00 ± 0.02, group II: 0.52 ± 0.05, *P* < 0.001; group III: 0.80 ± 0.04, *P* < 0.001; Fig. 5A). The TH protein level in the bilateral striatum was shown in Fig. S4. These findings suggested that US provided neuroprotective effects for DA neurons.

### Holographic US stimulation of the bilateral striatum prevented axon degeneration from MPTP toxicity

The stimulation targets were the bilateral DS, and then we ask why holographic US could exert treatment effect on the other region (the SN). Postmortem studies have suggested axon degeneration prior to the loss of neuronal soma in the brains of patients with neurodegenerative diseases, even in the absence of the clinical symptoms [42,43]. An in vivo study demonstrated that the reduction of DA axons in the striatum occurred earlier than the apoptosis of DA neurons in the SN in PD mouse model [44,45]. Thus, retarding axon degeneration could be a strategy to postpone the death of DA neurons. Previous studies have indicated that phosphorylated Akt (p-Akt) and phosphorylated CRMP2 (p-CRMP2) were involved in axon degeneration [46,47]. We found elevated levels of p-Akt and suppressed expression of p-CRMP2 in the striatum after MPTP injection, and holographic US treatment reversed this situation (p-CRMP2: group I: 1.00 ± 0.07, group II: 2.48 ± 0.39, *P* < 0.001; group III: 1.56 ± 0.14, *P* = 0.037, p-Akt: group I: 1.00 ± 0.02, group II: 0.38 ± 0.07, *P* < 0.001; group III: 0.71 ± 0.06, *P* = 0.001; Fig. 5A and B). β3-Tubulin is a neuron-specific cytoskeletal protein [48], and the expression of β3-tubulin was up-regulated after holographic US treatment compared with that in the MPTP + sham group mice (group I: 1.00 ± 0.07, group II: 0.55 ± 0.06, *P* < 0.001; group III: 0.82 ± 0.02, *P* < 0.001; Fig. 5E and F). Growth associated protein-43 (GAP-43) promotes nerve regeneration and synaptic connections [49]. The expression of GAP-43 was up-regulated after holographic US stimulation compared with the MPTP + sham group mice (group I: 1.00 ± 0.03, group II: 0.35 ± 0.06, *P* < 0.001; group III: 0.72 ± 0.04, *P* < 0.001; Fig. 5E and F). The protein levels of GAP-43, p-Akt, β3-tubulin, and p-CRMP2 in the SN were shown in Fig. S5. These data collectively demonstrated that holographic US stimulation of the bilateral DS prevented axon degeneration in PD mice.

### Holographic US stimulation of the bilateral striatum facilitated PSD formation in the DS

**Fig. 6.**
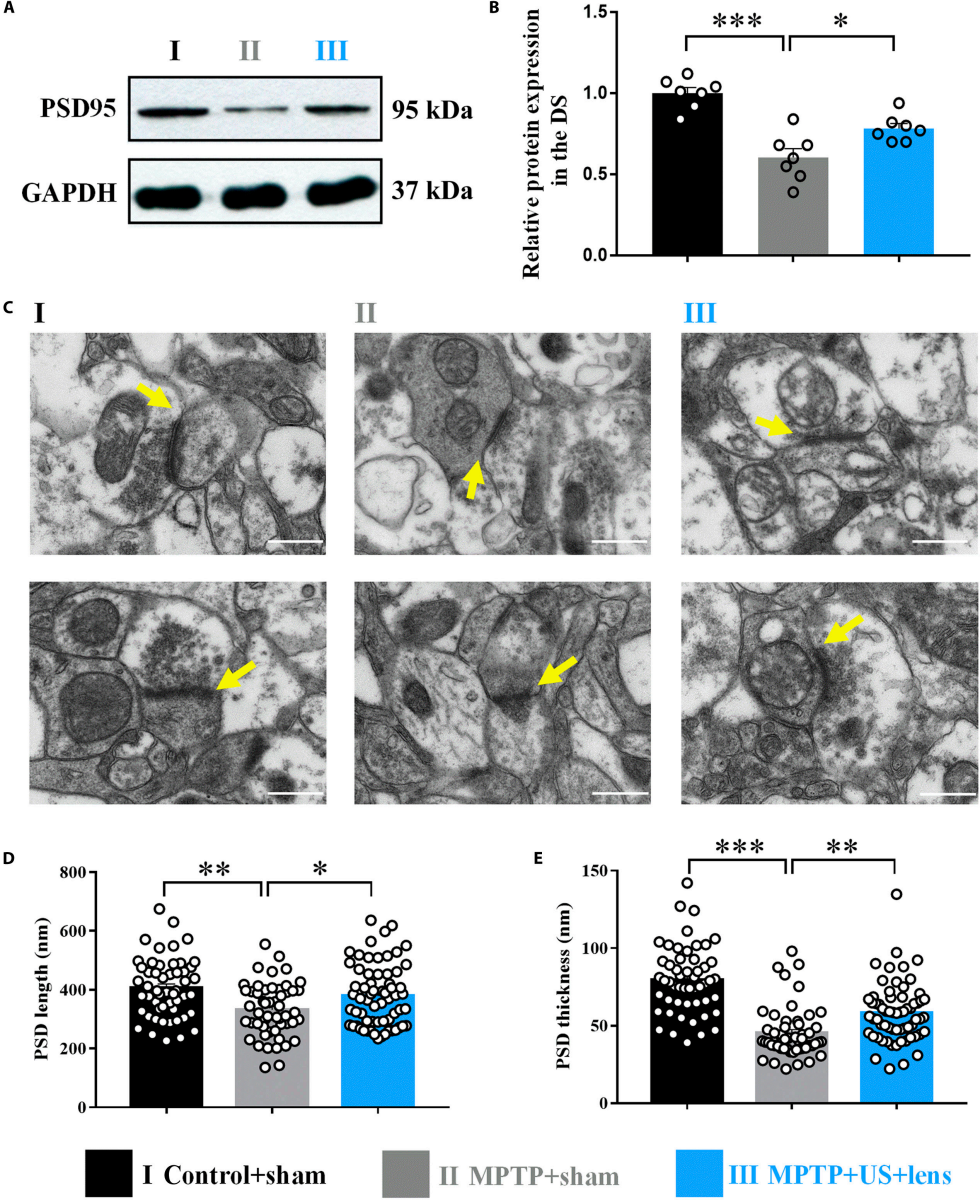
Holographic US facilitated PSD formation in the DS. (A and B) PSD95 was decreased in the DS after MPTP injection, which was increased using holographic US treatment. (C) EM images of the mice striatum (yellow arrows: PSD complex). Statistical graph for the PSD length (D) and thickness (E). One-way ANOVA with Tukey’s post hoc: *P < 0.05, **P < 0.01, ***P < 0.001; mean ± SEM, *n* = 7 for WB analysis; one-way ANOVA with Bonferroni’s post hoc, *n* = 52 synapses from 4 mice for groups I and II, *n* = 64 synapses from 4 mice for group III for EM detection. Scale bar, 500 nm.

Postsynaptic density protein 95 (PSD95), a structural anchoring protein, participates in synaptic transmission [50]. A previous study found that the motor performance increased in combination with elevated protein levels of PSD95 in the brain [51]. Conversely, the lack of PSD95 in mice would lead to motor impairment [52]. Thus, we examined the protein level of PSD95 using WB analysis, and the length and thickness of PSD95 in the DS using electron microscopy (EM) detection, to explore the effect of the holographic US stimulation on the spine and PSD95 formation. As shown in Fig. 6A, the protein levels of PSD95 were down-regulated after MPTP injection, but holographic US reversed this situation (group I: 1.00 ± 0.04, group II: 0.60 ± 0.06, *P* < 0.001, group III: 0.78 ± 0.03, *P* = 0.023; Fig. 6B). Consistent with the results, both the length and thickness of PSD95 in the DS were increased following holographic US (PSD length: group I: 405.82 ± 14.05, group II: 331.54 ± 12.60, *P* = 0.001, group III: 378.56 ± 13.21, *P* = 0.038; PSD thickness: group I: 79.42 ± 3.04, group II: 45.19 ± 2.29, *P* < 0.001, group III: 58.20 ± 2.37, *P* = 0.001). These results demonstrated that holographic US stimulation of the bilateral DS modulated spine density, potentiated synaptogenesis, and further improved the motor performance in PD mice.

## Discussion

In this study, we proposed a multi-target neuromodulation method and demonstrated holographic US treatment for mouse model of PD. We reconstructed the acoustic beams using Rayleigh–Sommerfeld and time-reversal methods. The holographic lens could generate bifocal beam, matching with the location of the left and right DS, as in the free space. The results suggested that: (a) holographic US stimulation could serve as a multi-target neurostimulation tool, (b) holographic US stimulation of the bilateral DS could ameliorate the motor symptoms induced by MPTP injection, and (c) the underlying mechanisms could be holographic US stimulation relieved the axon degeneration and facilitated the PSD formation in the striatum, which further provided neuroprotective effect for DA neurons and enhanced synaptic transmissions.

One of the challenges in achieving precise US neuromodulation is to overcome beam aberrations during transcranial propagation. Time-reversed invariance combined with phased arrays could retrieve the skull-aberration layers and produce multi-focus stimulation [53]. However, this required a large number of channels and incurred considerable economical costs [35]. Jiménez-Gambín et al. [37] produced acoustic beams with complex spatial distributions in human skull phantom using holographic lens. They did not conduct in vivo experiments to verify the flexibility of holographic transducers. In this study, we proposed a 3D-printed acoustic holographic lens (10 mm × 10 mm), which could combine with a wearable transducer to provide multi-target US stimulation for freely moving mice. The wearable transducer for freely moving mice could avoid the effect of anesthetics on behavior changes and neuronal cell death [54]. Besides, we would conduct multi-targets stimulation and explore the effect of real-time US stimulation on behavioral experiments.

The transducer used in this experiment was small and operated at high frequency, making it difficult to translate into human trials. The following approach should be considered when the method would be translated into the clinics. First, the skull or tissue’s attenuation on the acoustic wave increases with the rise in driving frequency, while the focal spot size decreases correspondingly. Given that, the target area in the human brain is significantly larger than that in mice. Transcranial focused US could operate at a lower driving frequency (below 1 MHz) for the human brain, which can not only enhance acoustic transmission but also ensure the acceptable precision required for brain stimulation. Second, high-resolution computed tomography (CT) scans could provide detailed information on the skull’s density and thickness distribution. Consequently, phase corrections could be calculated to counteract wave distortion caused by the skull, utilizing US propagation models. Third, the acoustic lens could be meticulously designed and crafted based on these calculated phase corrections. Furthermore, the integration of magnetic resonance imaging (MRI) for temperature imaging has revolutionized the guidance, monitoring, and control of focused US beams, significantly enhancing both the precision and safety of the procedure.

The main clinical symptoms of PD are motor dysfunctions, including tremor, rigidity, postural instability, and bradykinesia, which greatly influence the self-care ability. Thus, controlling the motor function is the priority aim for PD treatment. In this study, the behavioral performance verified that holographic US stimulation of the bilateral striatum improved the motor function. We also detected the treatment effect of unilateral US stimulation (Fig. S6). The behavioral test (Fig. S6B) indicated that unilateral US stimulation of the striatum could not improve the motor function in the rotarod. These results indicate that the therapeutic effect of bilateral US stimulation is superior to that of unilateral stimulation. These results are consistent with results from the bilateral DBS [55], which demonstrated that bilateral DBS provides significantly greater improvement in parkinsonism compared to unilateral DBS. In a previous study, we demonstrated that the subthalamic nucleus (STN) is an effective nucleus for US stimulation therapy in treating PD [56]. In the future work, we would make a comparison of the outcome between the STN and the striatum stimulation to explore the differences in efficacy between the 2 targets.

We further explored the molecular mechanisms underlying the holographic US stimulation of the DS. We chose the bilateral DS as the stimulation targets, so we investigated the changes in the axon and synapses to explore the underlying mechanism of the holographic US in PD mice. GAP-43, β3-tubulin, p-Akt, and p-CRMP2 are involved in axon degeneration in neurodegenerative diseases [57]. GAP-43 is involved in neuronal sprouting, and the expression of GAP-43 was decreased in the striatum of PD patients from postmortem tissue [58]. β3-Tubulin and p-Akt participate in axon guidance and maintenance [59]. The increased levels of p-CRMP2 promote axon degeneration [60]. In this study, the increased levels of GAP-43, β3-tubulin, and p-Akt and the decreased levels of p-CRMP2 indicated that the axon degeneration was improved after holographic US treatment of the bilateral striatum. These results indicated that the DS maybe a potential target for US neuromodulation of PD. The neuropathology of PD includes mitochondrial dysfunction, oxidative stress, neuro-inflammation, α-synuclein accumulation, and axonal transport [61]. In our previous studies, we found that US exerted anti-oxidative [56] and anti-apoptotic effects [31] and suppressed neuroinflammation [30] in PD mice. In the present study, we further revealed that US neuromodulation led to neural remodeling, including the remodeling of synapses and axons. Our studies also indicated that US neuromodulation provided a multifaceted treatment effect for PD.

The expression of c-Fos is widely used as a surrogate marker of neural activity [62]. The c-Fos-positive cells were increased in the DS after holographic US stimulation of the striatum, indicating that US specifically activated the basal ganglia circuit. Additionally, we assessed the safety of the holographic US stimulation. The corresponding mechanical index (MI) 0.5 and *I*_spta_ (spatial-peak temporal-average intensity) 180 mw/cm^2^ were considerably lower than the Food and Drug Administration (FDA) limits [63]. Moreover, hematoxylin and eosin and Nissl staining of the striatum (Fig. S7) revealed that the tested US parameters did not cause any tissue damage or neural loss in healthy mice. The PRF (500 Hz), which is far below the hearing frequency of mice (1 kHz), was adopted in our study to avoid potential auditory effect [31,64]. In our previous study [64], we found that shear waves traveling along the skull failed to achieve the necessary acoustic pressure in the auditory cortex to influence neuron activity. Additionally, we discovered that US stimulation (500-Hz PRF) of the ventral tegmental area (VTA) or the primary motor cortex (M1) could rouse deaf mice from anesthesia [36]. Furthermore, without US coupling gel to enhance the transmission of US wave into the brain nuclei, US failed to improve the mice behavioral performance [64]. We also suggested that 1-kHz PRF US stimulation of the STN, but not the primary visual cortex, diminished Parkinson’s-related symptoms [2]. These results demonstrated that behavioral changes were induced by US stimulation of the corresponding brain nuclei, and the activation of the auditory loop is not involved in the behavioral response.

In summary, we designed the holographic lens using Rayleigh–Sommerfeld and time-reversal method to construct multi-focus acoustic fields inside the mouse skull for neural circuit stimulation. We confirmed that the bilateral US stimulation of DS could improve the motor function of PD mice. Besides, holographic US provided neuroprotective effect for DA neurons by alleviating axon degeneration and promoting postsynaptic densities. Together, these studies demonstrated that the DS may serve as a novel target for ultrasonic treatment of PD.

## Materials and Methods

### Transducer fabrication

The assembly diagram of the holographic transducer is shown in Fig. 1B. We chose PZT-8 ceramic (a resonant frequency of 3 MHz, Siansonic) as the piezomaterial due to its high-quality factor and low dielectric loss. The negative electrode and positive electrode were on the small side of the PZT-8 ceramic. First, the stimulating electrode was connected with the positive and negative electrode on the back of the PZT-8 ceramic. Then, the holographic lens was coated to the PZT-8 ceramic using epoxy (Epo-Tek 301). Finally, the PZT-8 ceramic was placed into the transducer housing, and epoxy was casted to the back of the ceramic. A collimator (Fig. S8) was designed to fix the holographic transducer onto the certain region of the mouse skull. The bottom of the collimator was arc-shaped to fit the shape of the skull. The size of the holographic transducer was 10 mm × 10 mm × 2 mm, and the total weight was about 2 g.

### Acoustic parameters

The pulse parameters were as follows: FF 3 MHz, PRF 500 Hz, DC 20%, TBD 0.4 ms, SD 1 s, ISI 4 s. The pulse US wave was generated by a 2-channel waveform generator (Rigol, DG4162). Channel 1 was square wave with 1 s on and 4 s off, while channel 2 was 3-MHz sine wave with 0.4 ms on and 1.6 ms off. The signal for channel 1 was used to trigger channel 2, and the voltage from channel 2 was then driven by a 100-W amplifier (EI 2100L). The acoustic intensity field with/without holographic lens was measured by the US transmission technique (UMS3, Precision Acoustics, UK). A calibrated acoustic needle hydrophone with a diameter of 0.2 mm was used to collect the pulse US wave that passed through the holographic lens and mouse skull. Furthermore, the hydrophone was controlled by a stepper motor, and 2D scanning was performed over the *x*-*z* plane perpendicular to the holographic lens with a spatial resolution of 0.1 mm × 0.1 mm. In experimental measurement, *I*_spta_ after passing the mouse skull was 180 mW/cm^2^.

### Animals and experimental procedure

C57/BL6J mice (male, 8 weeks old, body weight 20 to 24 g) were used in the experiments. All animal procedure were approved by the Institutional Ethics Committee of Animals Experimentation (SIAT-IACUC-210308-YGS-NLL-A1745). All mice were anaesthetized and fixed on a stereotaxic apparatus. The scalp of each mouse was cut using scissors to expose the mouse skull. The location of the DS (relative to the bregma, +0.38 mm anterior/posterior, ± 2.3 mm medial/lateral) was marked on the skull using a pen. A cross line was used to guide the collimator installation. After installing the collimator, all mice rested for 5 d before proceeding with further experiments. For c-Fos expression (Fig. 3), all mice were divided into sham and holographic US group. For the sham group, the healthy mice wore the holographic transducer while the US power was turn off. For the holographic group, the healthy mice received bilateral US stimulation (3 MHz FF, 500 Hz PRF, 20% DC, 0.4 ms TBD, 1 s SD, 4 s ISI) of the DS for 30 min. For behavior and biological sample test (Figs. 4 to 6), all mice were divided into (I) control + sham, (II) MPTP + sham, and (III) MPTP + US + lens (holographic US stimulation) groups. During the preparation stage (day −6 to day 0), the group II and group III mice received intraperitoneal injection of MPTP (30 mg/kg, Sigma-Aldrich) to induce parkinsonism behavioral dysfunction. Meanwhile, the group I mice received an equivalent volume of saline. During the treatment stage (days 1 to 10), the group III mice received holographic US stimulation (3 MHz FF, 500 Hz PRF, 20% DC, 0.4 ms TBD, 1 s SD, 4 s ISI) for 10 d. The group I and group II mice wore the transducer with the US power turn off. For unilateral US stimulation, mice were divided into MPTP + sham and MPTP + unilateral US groups. US wave (3 MHz FF, 500 Hz PRF, 20% DC, 0.4 ms TBD, 1 s SD, 4 s ISI) was delivered to the striatum of the left hemisphere, and the rotarod test was adopted after 10 d of unilateral US stimulation. For unilateral US stimulation, the acoustic parameters (3 MHz FF, 500 Hz PRF, 20% DC, 0.4 ms TBD, 1 s SD, 4 s ISI, 180 mW/cm^2^ *I*_spta_) was the same with the parameters used for bilateral stimulation. The US wave was delivered to the left striatum 30 min for 10 d. We adopted a single-element focused transducer for unilateral stimulation, and the piezoelectric ceramic (PZT-8, Siansonic) was with 12 mm diameter and 12 mm radius of curvature. For bilateral stimulation, we combined the holographic lens with a wearable transducer, and the piezoelectric ceramic (PZT-8, Siansonic) was with 10 mm × 10 mm length and 1 mm height. The numerical acoustic field distribution was shown in Fig. S9.

### Behavioral assessment

The pole test and rotarod test were adopted to assess the motor function of PD mice, as previously described [31]. In the pole test, a pole with 50 cm height and 1 mm diameter was adopted to detect the bradykinesia and movement balance of PD mice. Mice were put on the top of the pole, and the time spent climbing down the pole was recorded. Each mouse was tested twice, and the average time was calculated as locomotor time for statistical analysis. In the rotarod test, mice were put on a rod with speed accelerated for 0 to 40 rotations per minute (rpm) in 5 min. Each mouse was placed on the rod, and the time stayed on the rod was defined as latency to falls. Each mouse was tested twice, and the average time was calculated for statistical analysis. The mice performance in the rotarod test reflected the motor coordination of PD mice.

### Immunohistochemistry staining

For c-Fos staining, the holographic US group mice received 30 min of holographic US stimulation. The sham group mice wore the holographic transducer while the transducer power was turn off. All mice were anaesthetized using isoflurane after 90-min rest and then perfused by phosphate-buffered saline (PBS) and 4% paraformaldehyde (PFA). The brain tissue containing the striatum was embedded in the paraffin, and 4-μm brain slices were obtained using a microtome (RM2016, Leica, Germany). For TH staining, brain tissue containing SN and striatum was embedded in paraffin and 4-μm coronal sections were obtained using a microtome (RM2016, Leica, Germany). All brain slices were incubated in the primary antibody (Table S1) at 4 °C overnight and processed with secondary antibody (Table S1) for 30 min at room temperature. Images were captured using a digital slide scanner (Pannoramic, 3D HISTECH). The c-Fos-positive neurons were manually tallied at 10× magnification, whereas the TH-positive neurons were tallied at 20× magnification by observers who were double blinded to the experimental conditions.

### Western blotting

The striatum and the midbrain containing the SN of the left hemisphere were dissected out on ice and stored at −80 °C. In the WB analysis, these samples were prepared in radioimmunoprecipitation assay buffer for 30 min, and tissue lysates were centrifuged at 12,000 rpm for 10 min. The membranes were incubated in the anti-TH (1:200), anti-GAP-43 (1:2,000), anti-p-Akt (1:500), anti-β3-tubulin (1:800), and anti-p-CRMP-2 (1:500) antibodies overnight at 4 °C. After that, membranes were washed and incubated in the goat anti-mouse immunoglobulin G (IgG) (1:5,000) or goat anti-rabbit IgG (1:5,000) antibodies at room temperature for 2 h. The intensities of the bands were normalized to glyceraldehyde-3-phosphate dehydrogenase (GAPDH) (ab181602, 1:3,000) levels and then normalized to the control + sham group.

### Electron microscopy

The SN and striatum of the right hemisphere received EM detection. Mouse tissues were blocked in a fixative reagent (G1102, Servicebio, Wuhan, China) at 4 °C for 2 h [51]. Tissues were fixed in 1% osmium tetroxide for 2 h and dehydrated in 50 to 100% graded ethanol solutions and 2 times of acetone (15 min each). Tissues were embedded in Embed 812 at 60 °C for 48 h (90529-77-4, Structure Probe Inc.) and cut into 80-nm slices. Slices were stained with uranyl acetate and lead citrate for 15 min each. Images were captured using a transmission electron microscope (Hitachi, Japan). The length and thickness of the PSD were counted under 46,000× magnification.

### Statistical analysis

Statistical analysis was conducted by SPSS statistics. Data were presented as mean ± standard error of the mean (SEM). For 2-groups comparison, independent-sample *t* test was conducted. For multiple groups comparison, one-way analysis of variance (ANOVA) followed by Tukey’s post hoc was conducted when the sample size for each group was equal. Bonferroni’s post hoc was conducted when the group sample sizes were unequal. Statistical significance was set at *P < 0.05, **P < 0.01, and ***P < 0.001.

## Data Availability

All data needed to support the conclusions of this work are available within the paper and the Supplementary Materials.
