# Supplementary material for: Holographic Ultrasound Modulates Neural Activity in a 1-Methyl-4-Phenyl-1,2,3,6-Tetrahydropyridine-Induced Mouse Model of Parkinson’s Disease
Source: Research (Wash D C). 2024 Nov 6;7:0516. doi: 10.34133/research.0516 (PMC11538569; doi:10.34133/research.0516)
Supplement: Supplementary 1 — Figs. S1 to S9 Table S1 [file research.0516.f1.zip › supplementary file.docx]

**Supplementary Information**

Holographic Ultrasound Modulates Neural Activity in an MPTP-induced Mouse Model of Parkinson's Disease

Hui Zhou^1,2†^ , Fei Li^1†^, Zhengrong Lin^1^, Long Meng^1^, Dan Chen^3^, Qingping Zhang^4^, Lili Niu^1*^

^1^Paul C. Lauterbur Research Center for Biomedical Imaging, Institute of Biomedical and Health Engineering, Shenzhen Institutes of Advanced Technology, Chinese Academy of Sciences

^2^Tech X Academy, Shenzhen Polytechnic University, Shenzhen

^3^Institute of Ultrasonic Technology, Institute of Intelligent Manufacturing Technology, Shenzhen Polytechnic University, Shenzhen

^4^School of Electronic and Communication Engineering, Shenzhen Polytechnic University, Shenzhen

†These authors contributed equally to this work and are co-first authors

Correspondence e-mail: [ll.niu@siat.ac.cn](mailto:ll.niu@siat.ac.cn)


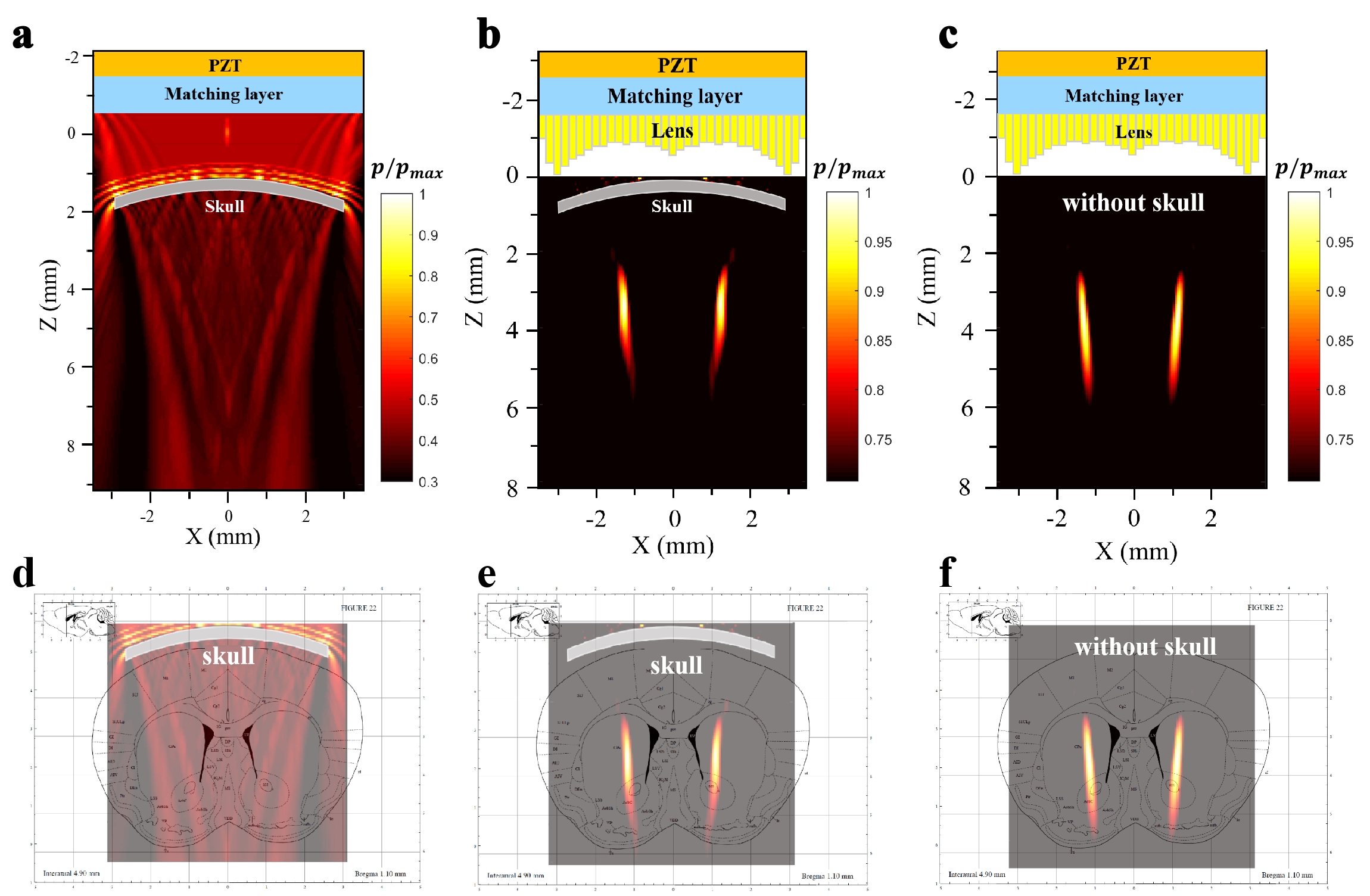


**Supplementary Fig. 1.** Numerical simulated pressure field of the transducer without lens (a), numerical simulated pressure field of the holographic transducer after passing the skull (b), and numerical simulated pressure field of the holographic transducer without skull (c). The corresponding overlap of the striatum and pressure field without lens (d). The corresponding overlap of the striatum and pressure field with lens after passing mouse skull (e). The corresponding overlap of the striatum and pressure field with lens without mouse skull (f).


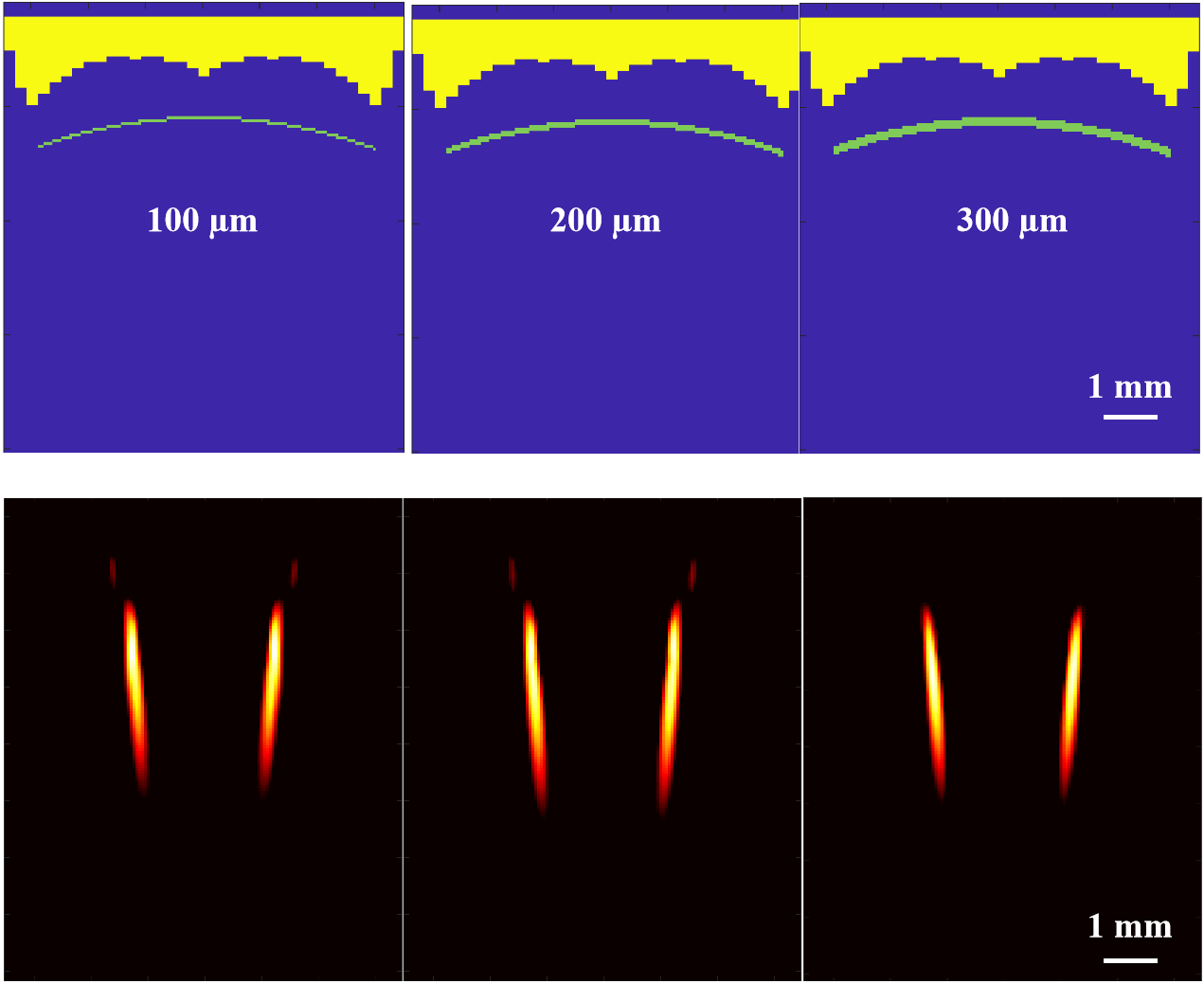


**Supplementary Fig. 2. Numerical simulated pressure distributions with skulls thicknesses ranging from 100µm to 300µm.**


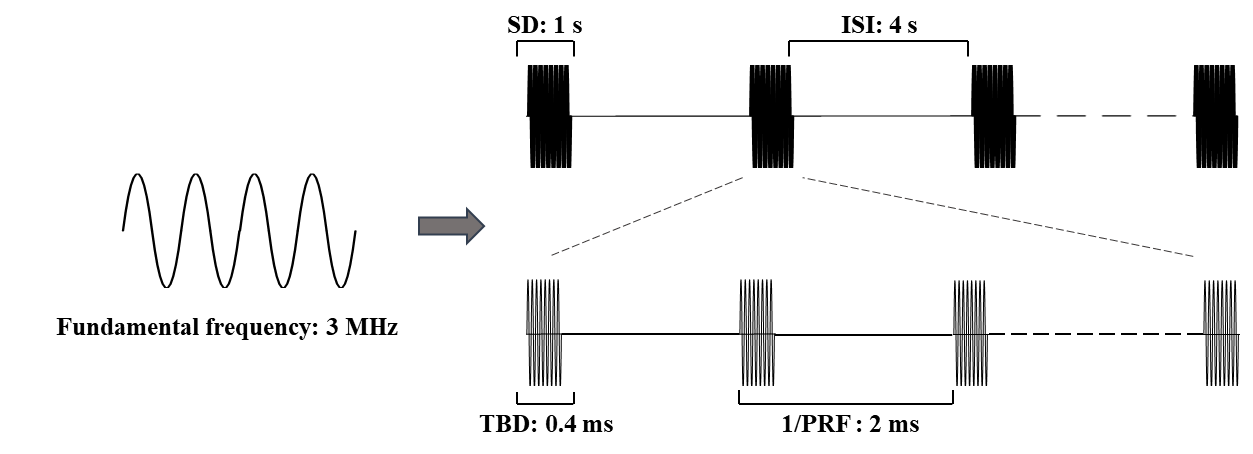


**Supplementary Fig. 3. The holographic ultrasound wave used in the experiment.** The ultrasound parameter was as follows: fundamental frequency: 3 MHz, pulse repetition frequency: 500 Hz, duty cycle: 20%, tone-burst duration: 0.4 ms, sonication duration: 1 s, inter-stimulus interval: 4 s.


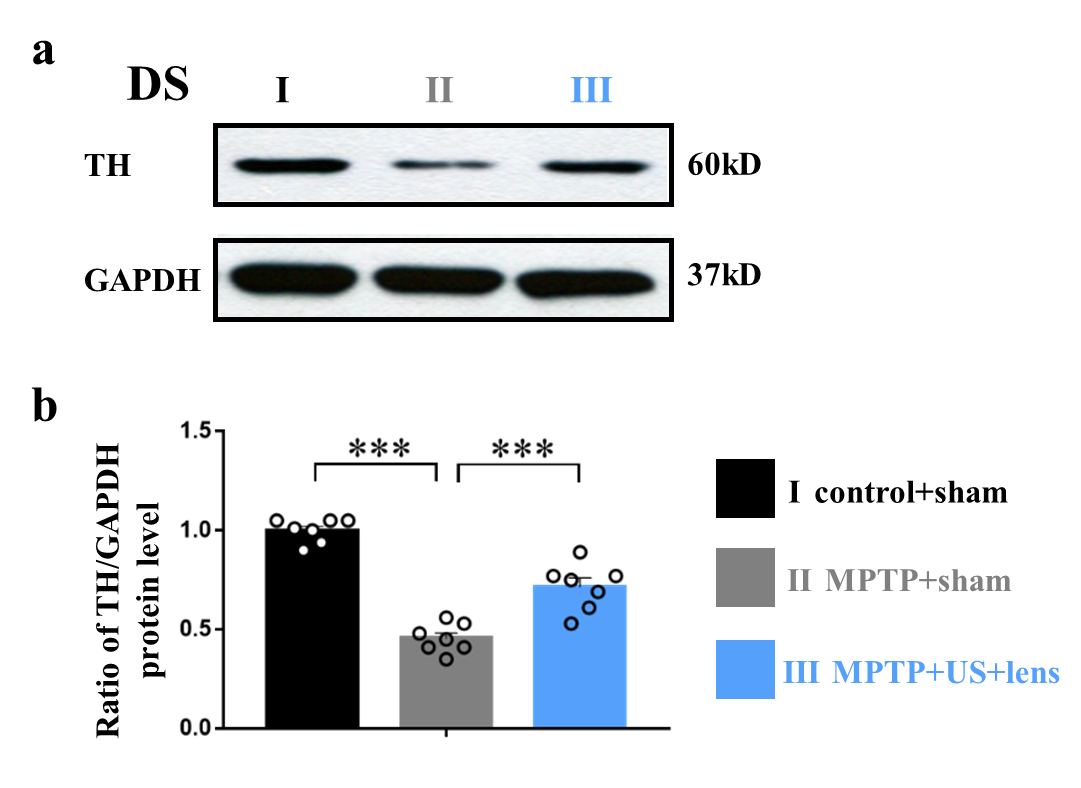


**Supplementary Fig. 4.** **Holographic ultrasound provided neuroprotective effect in the mouse dorsal striatum.** (**a**) Representative western blotting bands from the dorsal striatum. (**b**) Quantification of protein expression in three groups. (one–way ANOVA with Tukey’s post hoc: *p < 0.05, **p < 0.01, ***p < 0.001; mean ± SEM, n = 7, group Ⅰ: 1.00 ± 0.02, group Ⅱ: 0.46 ± 0.03, p < 0.001, group Ⅲ: 0.72 ± 0.05, p < 0.001)

**
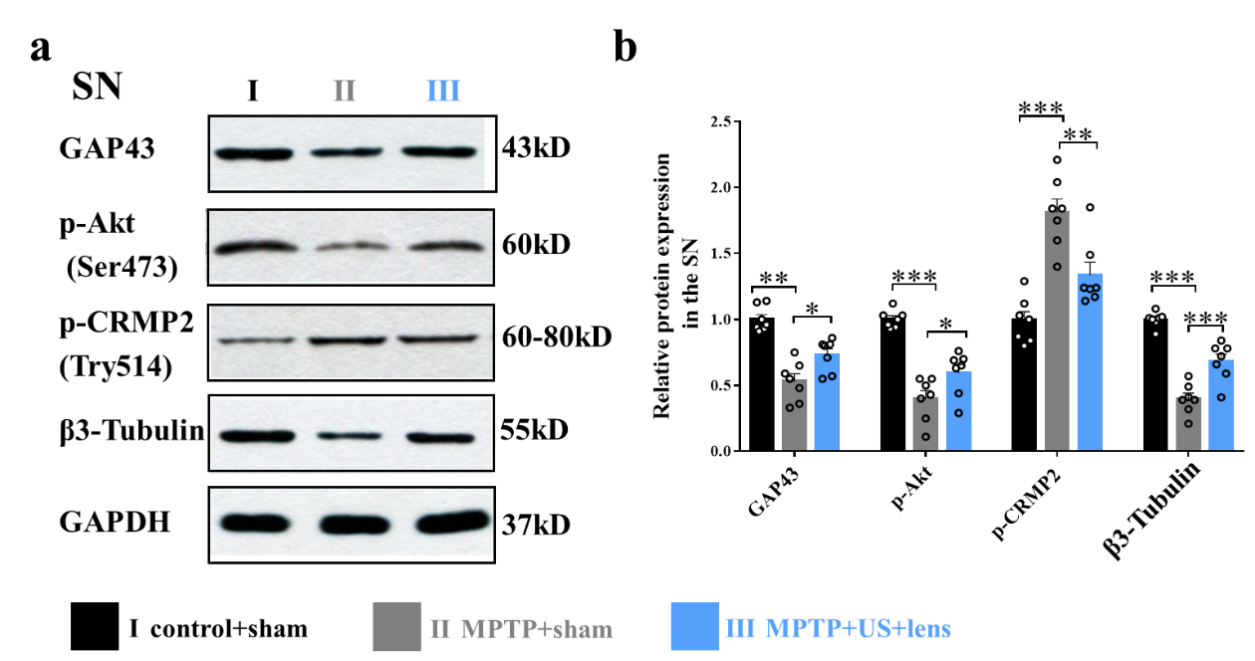
**

**Supplementary Fig. 5.** **Holographic ultrasound provided neuroprotective effect against the axon degeneration.** (**a**) Representative western blotting bands from the striatum. (**b**) Quantification of protein expression in three groups. (one–way ANOVA with Tukey’s post hoc: *p < 0.05, **p < 0.01, ***p < 0.001; mean ± SEM, n = 7; GAP43: group Ⅰ:1.00 ± 0.04, group Ⅱ: 0.53 ± 0.06, p < 0.001; group Ⅲ: 0.73 ± 0.05, p = 0.023; p-CRMP-2: group Ⅰ:1.00 ± 0.07, group Ⅱ: 1.81 ± 0.10 p < 0.001; group Ⅲ: 1.34 ± 0.10, p = 0.004; p-AKT: group Ⅰ:1.00 ± 0.02, group Ⅱ: 0.40 ± 0.06 p < 0.001; group Ⅲ: 0.59 ± 0.06, p = 0.046; β-tubulin3: group Ⅰ:1.00 ± 0.02, group Ⅱ: 0.40 ± 0.04 p < 0.001; group Ⅲ: 0.68 ± 0.06, p < 0.001)


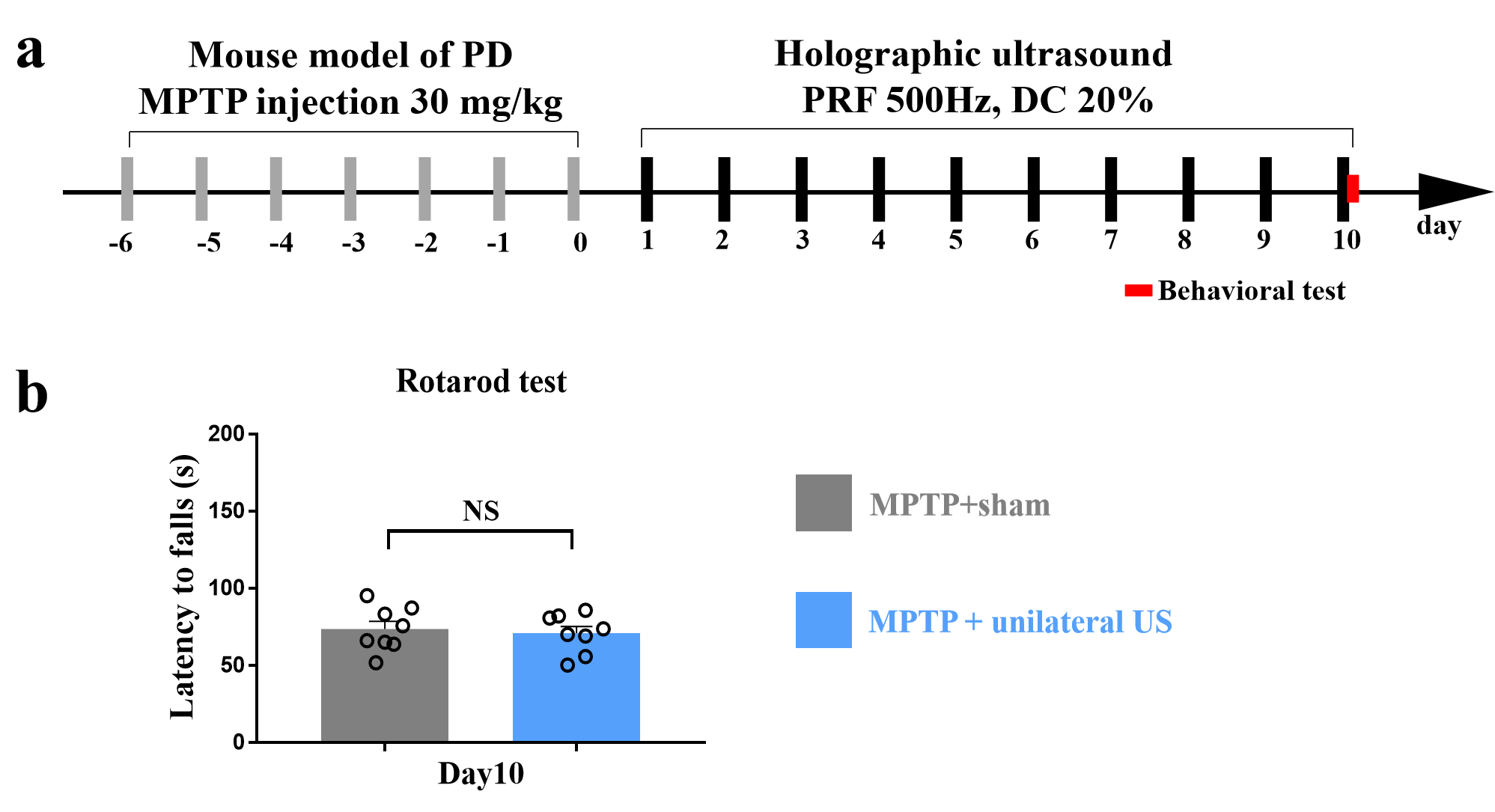


**Supplementary Fig. 6.** Unilateral ultrasound stimulation of the striatum did not improve the motor function in PD mice. (a) Illustration of behavior test protocol. (b) Unilateral ultrasound stimulation did not recover the latency to falls in the rotarod test. There was no obvious difference between MPTP + sham and MPTP + unilateral US group. (Independent-sample t-test, mean ± SEM, n = 8 each group)


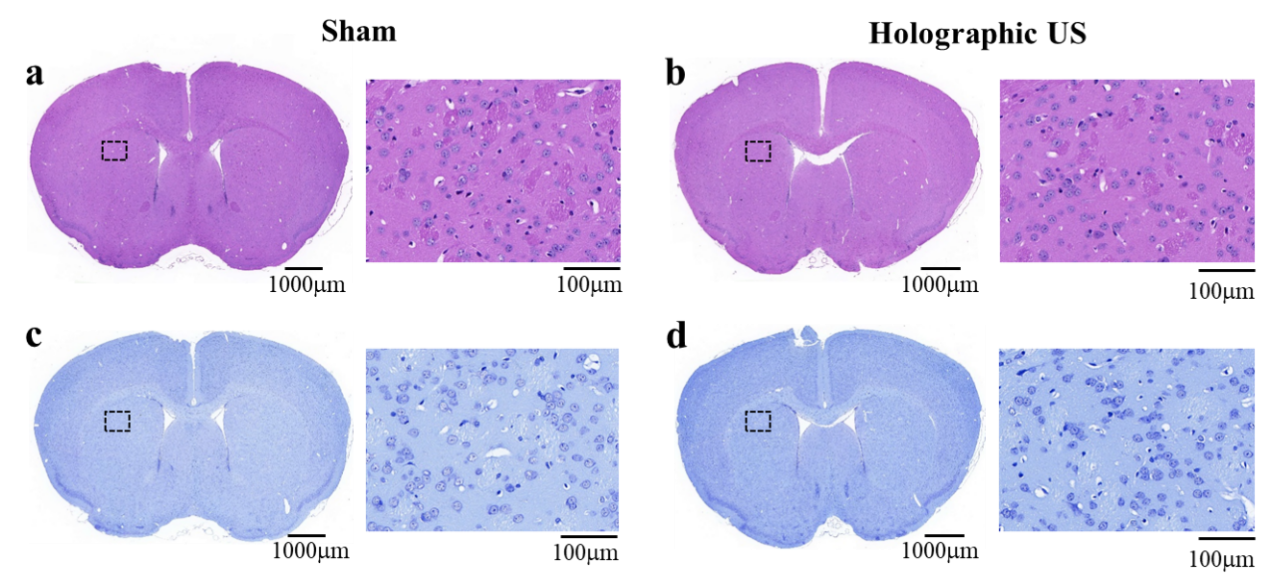


**Supplementary Fig. 7.** **Histological evaluation in the striatum after holographic ultrasound stimulation.** Representative HE staining (a, b) and Nissl (c, d) staining indicated no tissue damage caused by ultrasound stimulation


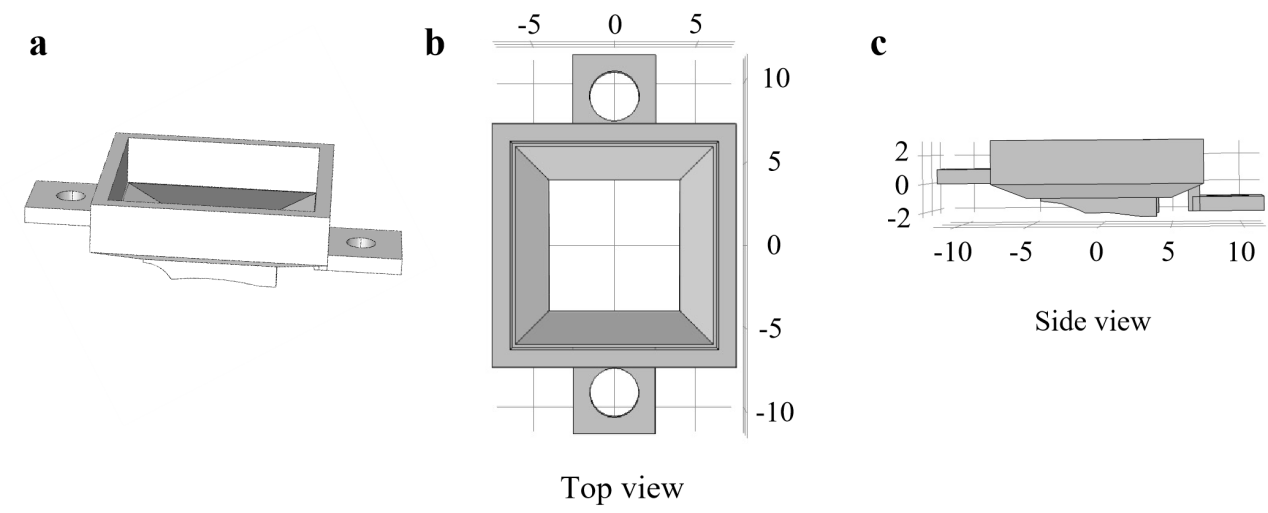


**Supplementary Fig. 8.** **The design of collimator** (**a), top view (b**) **and side view (c)**


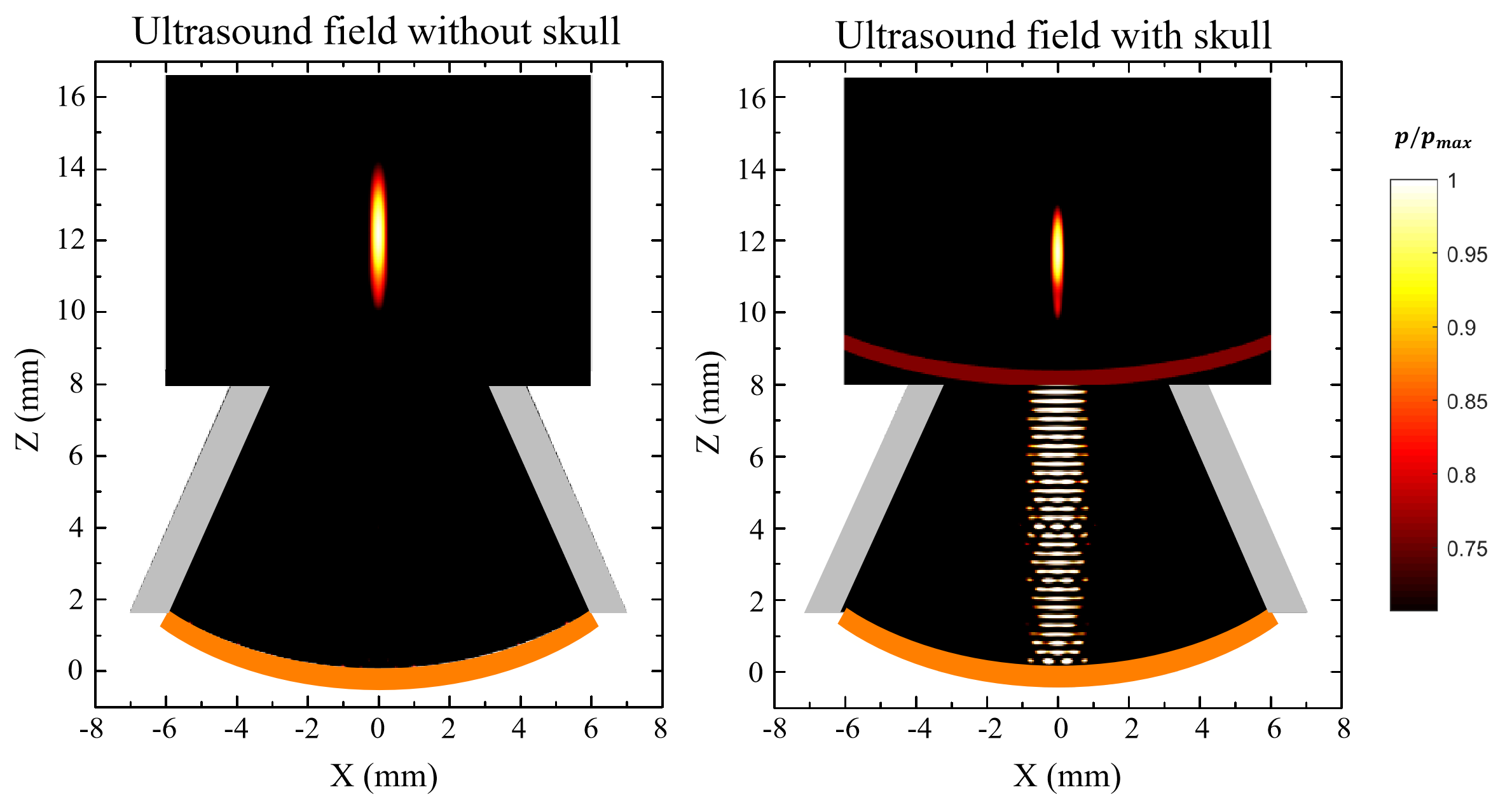


**Supplementary Fig.9. Numerically simulated pressure fields without (left) and with (right) mouse skull in unilateral ultrasound stimulation**

#### **Supplementary Table 1 Primary and secondary antibody used in western blotting and Immunohistochemistry**

|  | Primary antibody | Host | Company | Dilution | Catalog # |
| --- | --- | --- | --- | --- | --- |
| WB | TH | Rabbit | Abcam | 1:200 | 112 |
|  | GAP43 | Rabbit | Abcam | 1:2000 | 16053 |
|  | p-Akt | Rabbit | CST | 1:500 | 4060 |
|  | β3-Tubulin | Rabbit | CST | 1:800 | 5568 |
|  | p-CRMP-2 | Rabbit | CST | 1:500 | 9397 |
| IHC | TH | Rabbit | Abcam | 1:750 | 112 |
|  | c-Fos | Mouse | Abcam | 1:1000 | 208942 |
| Secondary  antibody (WB) | Goat-Anti-Mouse IgG, HRP | - | Abcam | 1:5000 | 6789 |
|  | Goat-Anti-Rabbit IgG, HRP | - | Abcam | 1:5000 | 6721 |
| Secondary  antibody (IHC) | Goat-Anti-Mouse IgG, HRP | - | Servicebio | 1:200 | 23301 |
|  | Goat-Anti-Rabbit IgG, HRP | - | Servicebio | 1:200 | 23303 |
